# Evaluation of quality of life in lung cancer patients receiving radiation and *Viscum album* L.: a real-world data study

**DOI:** 10.1186/s13014-023-02234-3

**Published:** 2023-03-06

**Authors:** Friedemann Schad, Diana Steinmann, Shiao Li Oei, Anja Thronicke, Christian Grah

**Affiliations:** 1 Hospital Gemeinschaftskrankenhaus Havelhöhe, Research Institute Havelhöhe, Kladower Damm 221, 14089 Berlin, Germany; 2 Interdisciplinary Oncology and Palliative Care, Hospital Gemeinschaftskrankenhaus Havelhöhe, Berlin, Germany; 3 Department of Radiotherapy and Special Oncology, Hannover Medical School, Hannover, Germany; 4 Lung Cancer Centre, Hospital Gemeinschaftskrankenhaus Havelhöhe, Berlin, Germany

**Keywords:** Integrative oncology, Lung cancer, Non-small-cell lung cancer, Quality of life, Mistletoe, Multimodal therapy, Radiotherapy

## Abstract

**Background:**

Lung cancer (LC) is associated with high mortality and poor quality of life (QoL). The disease as well as oncological treatments such as radiation and chemotherapy with adverse effects can impair the QoL of patients. Add-on treatment with extracts of *Viscum album* L. (white-berry European mistletoe, VA) has been shown to be feasible and safe and to improve the QoL of cancer patients. The aim of this study was to analyze the changes in QoL of LC patients being treated with radiation according to oncological guidelines and add-on VA treatment in a real-world setting.

**Methods:**

A real-world data study was conducted using registry data. Self-reported QoL was assessed by the evaluation of the European Organization of Research and Treatment Health-Related Quality of Life Core Questionnaire scale (EORTC QLQ-C30). Adjusted multivariate linear regression analyses were performed to analyze factors associated with changes in QoL at 12 months.

**Results:**

A total of 112 primary LC patients (all stages, 92% non-small-cell lung cancer, median age 70 (ICR: 63–75)), answered the questionnaires at first diagnosis and 12 months later. Assessment of 12 months changes in QoL revealed significant improvement of 27 points for pain (p = 0.006) and 17 points for nausea/vomiting (p = 0.005) in patients who received combined radiation and VA. In addition, significant improvements of 15 to 21 points for role (p = 0.03), physical (p = 0.02), cognitive (p = 0.04), and social functioning (p = 0.04) were observed in guideline treated patients receiving no radiation but add-on VA.

**Conclusions:**

Add-on VA therapy reveals supportive effects for the QoL of LC patients. Particularly in combination with radiation a significant reduction in pain and nausea/ vomiting has been observed.

*Trial registration* The study received ethics approval and was retrospectively registered (DRKS00013335 on 27/11/2017).

## Introduction

Lung cancer (LC) remains the leading cause of cancer deaths worldwide [1] and is often associated with a poor quality of life (QoL). Due to advances in treatment options for LC patients, the number of long-term survivors is growing, and their QoL is becoming increasingly important. Disease-related impairments in QoL are reported in the emotional, physical, social, and cognitive domains, as well as the activities of cancer patients in their daily living [2]. It was found that deterioration in physical functioning often persisted beyond two years after LC diagnosis, with pain, fatigue, dyspnea, and cough being the most common and distressing symptoms [3]. Radiation therapy is applied at all stages of LC. Current guidelines [4] do not recommend radiotherapy in stage I, II after R0 resection. For unresectable stage I cases, stereotactic radiotherapy or definitive radio (chemo) therapy is recommended depending on the affected lymph nodes, the patient's performance, and comorbidities. For primary non-resectable tumors in stage II-IIIB, neoadjuvant or definitive radio-(chemo-) therapy with subsequent resection, if possible, is recommended. In stage IV, radiotherapy can be applied for palliation of symptoms like upper venous congestion or as therapy of brain metastases. In patients with advanced LC (stage III and IV), early initiation of the radiation therapy may improve their QoL. In a prospective study with 164 patients with non–small-cell lung cancer (NSCLC) receiving radical radiotherapy, palliation of respiratory symptoms and improved QoL was observed [5]. Evaluations of patients with various cancer entities treated with palliative radiotherapy for brain metastases revealed a deterioration in QoL three months after radiation [6, 7]. The aim of integrative cancer therapy is to complement standard-oncological therapies such as surgery, chemotherapy or radiation by alleviating side effects and thereby also to improve QoL. Limiting the number and severity of disease symptoms, such as pain and dyspnea, and providing early psychological, social, and spiritual support have been shown to be critical for improving the QoL of LC patients [8], thus indicating that early palliative care may be supportive in cancer treatment strategies. As part of an integrative concept, *Viscum album* L. (VA) extracts are prescribed and utilized in German-speaking European countries to improve the QoL of cancer patients [9, 10]. In a previous real-world data (RWD) study we found, that the risk of death was reduced in patients with tumor stage IV NSCLC treated with a combination of chemotherapy and VA, compared to chemotherapy alone [11]. Only few publications so far have addressed self-reported QoL in LC patients [2, 3, 12–14]. In a further RWD study, our group has shown that LC patients, age-dependently or tumor stage-dependently report increased pain, low mood and financial difficulties at diagnosis before treatment [14]. Little is known about integrative treatments including VA extracts with respect to self-reported QoL in patients with LC and in most clinical LC trials QoL is only observed as a secondary outcome parameter [15, 16]. In addition, QoL data in patients with radio-chemotherapy and add-on VA treatment in oncological patients is in its infancy and primarily concentrated on patients with colorectal cancer [17] or rectal cancer in a neoadjuvant setting [18]. The present longitudinal RWD study investigated the self-reported QoL in guideline-treated LC patients and its associations with additional VA treatment applied alone or in combination with radiation.

## Methods

### Study design and patients

We conducted a longitudinal monocentric RWD study by extracting and analyzing demographic data, information on diagnosis, histology, integrative oncological treatment data as well as QoL data from the oncological registry Network Oncology (NO) [19]. Primary all-stage LC patients were included from whom written informed consent has been obtained. Surveys were conducted at diagnosis as well as 12 months after diagnosis using the European Organization of Research and Treatment Health-Related Quality of Life Core Questionnaire (EORTC QLQ-C30).

### Objective

The objective of this study was to analyze the self-reported QoL in guideline-treated LC patients in a LC center and the association with additional VA therapy applied alone or in combination with radiation.

### Data collection

As described in detail previously [20], patients with a histologically proven primary diagnosis of LC, who gave written consent, seen and treated at the Lung Cancer Center GKH in Berlin, Germany (certified according to the German Cancer Society since 2017) were screened. Patients were enrolled in the study from which assessable QoL data-sets at least at first diagnoses (T0) and 12 months later (T1) were available. The details of Union for International Cancer Care (UICC) cancer tumor stage, received surgery, radiation, and all treatment regimens were retrieved from the NO registry. All data reported here are based on retrievable data from the NO registry at cut-off date of July 15, 2021.

### Analyses of self-reported QoL

For the explorative evaluation of self-reported QoL the EORTC QLQ-C30 questionnaire was utilized and analyzed which includes evaluations of global health, functioning and symptom scales [21]. The EORTC QLQ-C30 questionnaires were assessed after first diagnosis (T0) and 12 months later (T1). Analysis of all 15 scales was performed as described in the EORTC QLQ-C30 manual [22]. The EORTC QLQ-C30 scores range from 0 to 100. Higher scores represent a better self-reported level for the functioning scale and a higher burden for the symptom scale, respectively.

### Statistical analysis

Demographic and diagnostic variables were collected at T0. Continuous variables were described as median with interquartile range (IQR); categorical variables were summarized as frequencies and percentages. Student´s t-tests were applied, to detect differences; p-values < 0.05 were considered to be significant. Multivariable linear regression analyses were performed to identify influencing factors and to address potential sources of bias and confounders. In order to yield reliable model results, stepwise regression selections were performed and models with high adjusted *R*^*2*^ were chosen. According to Cohen’s interpretation [23] *R*^*2*^ values between 0.13 and 0.25 indicate medium and *R*^*2*^ values 0.26 or above indicate high effect sizes. Predicting variables (with regard to T0) were age (in years), date of first diagnosis (in years), gender, EORTC QLQ-C30 values at T0 and for received treatments regimen rank-ordered variables were assigned (no treatment, 0; treatment, 1). For radiation (Rad) and VA treatments (VA) patients were categorized as VA (VA treatments only but no radiation), Rad (Rad only but no VA), and RadVA (Rad combined with VA), and patients who received neither Rad nor VA served as the reference. p-values < 0.05 were considered to be significant. All statistical analyses were performed using the software R (R Version 3.1.2 (2014)) [24].

## Results

### Patient’s characteristics

**Fig. 1:**
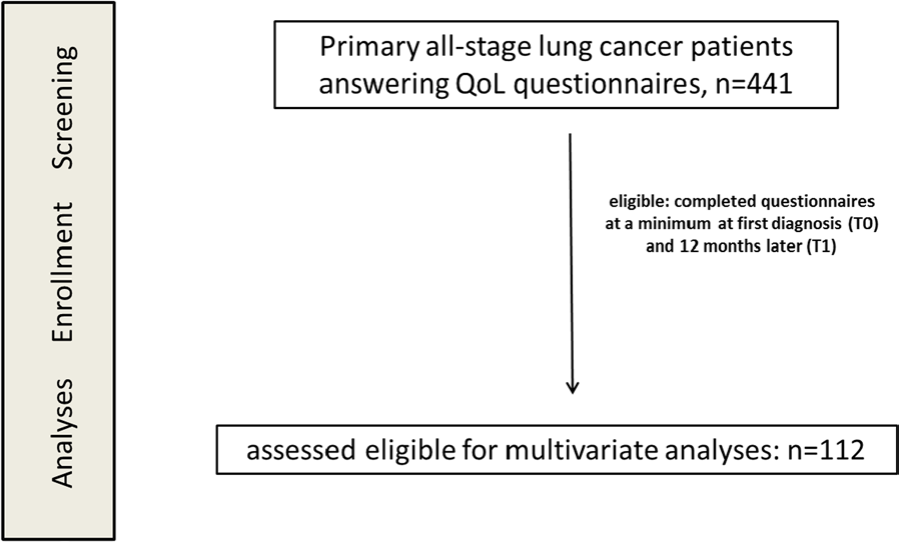
Flow chart of the study population. QoL, quality of life

In total, 441 all-stage LC patients treated between 2013 and 2021 at the certified German LC center answered EORTCT QLQ-C30 questionnaires at different time points. Eligibility for analysis was characterized by the availability of assessable data sets at a minimum at first diagnosis (T0) and 12 months later (T1). 131 patients died before T1, 49 LC patients were still under follow-up at cut-off day, and 112 patients answered QoL questionnaires at T0 and T1 (Fig. 1).

For 112 LC patients complete data were retrieved from the NO registry. In addition to standard guideline oncological systemic therapy, 53 (47%) patients received VA applications, administered at a median of 43 days (ICR: 23—74 days) after first diagnosis. For longitudinal analyses of treatment regimens, the 112 patients were allocated to 4 different groups according to the therapies they received within the 12 months. 27 patients receiving neither Rad nor VA served as reference group, 29 patients receiving VA applications without Rad were allocated to the VA group, 32 patients receiving radiation without VA were allocated to the Rad group, and 24 patients received Rad and VA and were allocated to the RadVA group. In Table 1, the main characteristics of analyzed patients are given for the entire study cohort and the groups separately. Major differences in age, gender, histology, and UICC stages were found between the groups. Patients receiving VA tended to be younger, in the VA group (p = 0.119), the majority was women (p = 0.016*), patients with histology other than NSCLC received significantly more RadVA (p = 0.042*), and patients with severe UICC tumor stages receiving significantly more VA (p = 0.0004*) or RadVA (p = 0.0018*) respectively. Following the recommendations of a multidisciplinary tumor board, all patients were treated according to guidelines. The majority of patients with UICC tumor stages I-III and one-third of patients with tumor stage IV underwent surgery. In Table 2 all treatments received within the observation period of 12 months are listed for the entire study cohort.

**Table 1:** Baseline characteristics of lung cancer patients

	Total n = 112 (100%)	Reference n = 27 (24%)	VA n = 29 (26%)	Rad n = 32 (29%)	RadVA n = 24 (21%)
*Sex*					
Men, n (%)	50 (45)	15 (56)	6 (21)	17 (53)	12 (50)
Women, n (%)	62 (55)	12 (44)	23 (79)	15 (47)	12 (50)
			p = 0.016*	p = 1	p = 0.908
*Age, years*					
Median (IQR)	70 (63–75)	71 (64–76)	65 (58–74)	72 (67–76)	68 (62–75)
			p = 0.119	p = 0.659	p = 0.363
*Histology*					
Non-small cell LC (NSCLC)	103 (92)	25 (93)	29 (100)	31 (97)	18 (75)
Small cell LC (SCLC)	6 (5)	0	0	1 (3)	5 (21)
Other#	3 (3)	2 (7)	0	0	1 (4)
			p = 0.328	p = 0.198	p = 0.042*
*UICC tumor stage*					
I	23 (21)	11 (41)	2 (7)	10 (31)	0
II	13 (12)	6 (22)	2 (7)	5 (16)	0
III	30 (27)	6 (22)	6 (21)	10 (31)	8 (33)
IV	46 (41)	4 (15)	19 (66)	7 (22)	6 (67)
			p = 0.0004*	p = 0.672	p = 0.0018*

*UICC* Union for International Cancer Care; *IQR* Interquartile Range

Significant p-values are indicated: *p-value < 0.05

#Other: one spindle cell carcinoid, two carcinoids without further specification

**Table 2:** Treatments of lung cancer patients

Treatments n = 112 (100%)	N (%)
**Surgery, n (%) 62 (55)**	**62 (100)**
Elective	60 (97)
Revision	3 (5)
Emergency	2 (3)
**Radiation (Rad), n (%) 56 (50)**	**56 (100)**
Thorax	45 (80)
Brain	14 (25)
Skeleton	7 (13)
**Chemotherapy, n (%) 69 (62)**	**69 (100)**
Platinum compounds	58 (84)
Vinorelbine	32 (46)
Pemetrexed	18 (26)
Taxanes	16 (23)
Etoposide	9 (13)
Gemicitabine	2 (3)
Bisphosphonates	11 (16)
**Targeted, n (%) 47 (42)**	**47 (100)**
Kinase inhibitors	15 (32)
Immune checkpoint inhibitors	33 (70)
*Viscum album L*. **(VA), n (%) 53 (47)**	**53 (100)**
Subcutaneous	49 (92)
Intravenous	35 (66)
Intra-tumoural/-pleural	3 (6)

The numbers in rows and columns of treatments applied to patients do not necessarily add to one hundred percent as patients may have received various combinations of preparations. *N* numbers

### Evaluation of longitudinal EORTC QLQ-C30 changes

Completed EORTC QLQ-C30 questionnaires were evaluated and analyzed for the entire study cohort at T0 and T1. All EORTC QLQ-C30 scores at T0 were within the range of formerly published EORTC QLQ-C30 reference values for LC patients [25] and similar as published previously [14]. In Table 3, the mean values for T0 and T1 for all EORTC QLQ-C30 scales and the means and IQR of the longitudinal changes for all scales were determined. Student’s t-test calculations revealed significant but not clinical impacting longitudinal improvements for global health (6.5 points; p = 0.006) and emotional functioning (7.6 points; p = 0.006).

**Table 3:** EORTC QLQ-C30 questionnaires of the entire study cohort at first diagnosis (T0) and 12 months thereafter (T1)

EORTC QLQ-C30	Key	T0		T1		T0–> T1		
n = 112		Mean	SD	Mean	SD	Changes	IQR	p-values
Global health status/QoL	QL	47.92	21.40	54.28	24.43	**6.46**	− 8.3 to 16.7	0.006*
Physical functioning	PF	62.70	24.79	58.15	24.95	− 4.56	− 20.0 to 13.3	0.063
Role functioning	RF	54.35	34.57	52.23	34.47	− 2.25	− 16.7 to 16.7	0.531
Emotional functioning	EF	50.61	22.55	58.04	28.30	**7.58**	− 8.3 to 25.0	0.006*
Cognitive functioning	CF	59.70	27.00	57.59	28.25	− 1.82	− 16.7 to 16.7	0.495
Social functioning	SF	60.49	30.81	57.89	32.27	− 3.40	− 16.7 to 16.7	0.269
Fatigue	FA	52.83	27.39	53.87	26.15	0.91	− 11.1 to 11.1	0.737
Nausea and vomiting	NV	10.06	19.41	10.42	20.79	0.45	0–0	0.845
Pain	PA	37.74	35.56	38.39	33.98	0.63	− 16.7 to 33.3	0.860
Dyspnea	DY	52.73	37.97	54.65	36.57	1.53	− 33.3 to 33.3	0.680
Insomnia	SL	47.04	36.73	42.26	37.79	− 5.61	− 33.3 to 33.3	0.171
Appetite loss	AP	38.84	35.12	30.36	37.68	**− 9.17**	− 33.3 to 0	0.015*
Constipation	CO	26.67	37.01	21.32	32.51	− 5.20	− 33.3 to 0	0.159
Diarrhea	DI	13.03	26.63	15.32	25.23	2.45	0–0	0.426
Financial difficulties	FI	20.32	29.28	27.38	30.61	**6.98**	0–33.3	0.01*

*EORTC QLQ-C30* European Organisation for Research and Treatment of Cancer quality-of-life questionnaire; *IQR* Interquartile range; *n* number of patients; *SD* standard deviation. p-values (two-sided, paired t-test) for longitudinal changes between T0 and T1

Significant p-values are indicated: *p-value < 0.05

For the entire study cohort, appetite was clinically meaningful and significantly improved (9.2 points; p = 0.015) while financial difficulties were aggravated (7 points; p = 0.01) (Table 3).

Multivariate linear regression analyses were performed for all EORTC QLQ-C30 scale changes with age, gender, year of LC diagnosis, and targeted therapy received as confounding variables. Since the EORTC QLQ-C30 scales at T0 varied highly (Table 3) they were used in addition as a continuous variable for all the multivariate analyses (Table 4). Patients receiving VA treatment without radiation were found to have considerable improvements in four functional and eight symptom scales (Table 4). These patients experienced significant and clinically relevant improvements in physical (estimate β = 16.1 ± 6.9; p = 0.02), role (estimate β = 21.2 ± 9.6; p = 0.03), cognitive (estimate β = 15.7 ± 7.6; p = 0.04), and social (estimate β = 17.0 ± 8.4; p = 0.04) functioning.

**Table 4:**
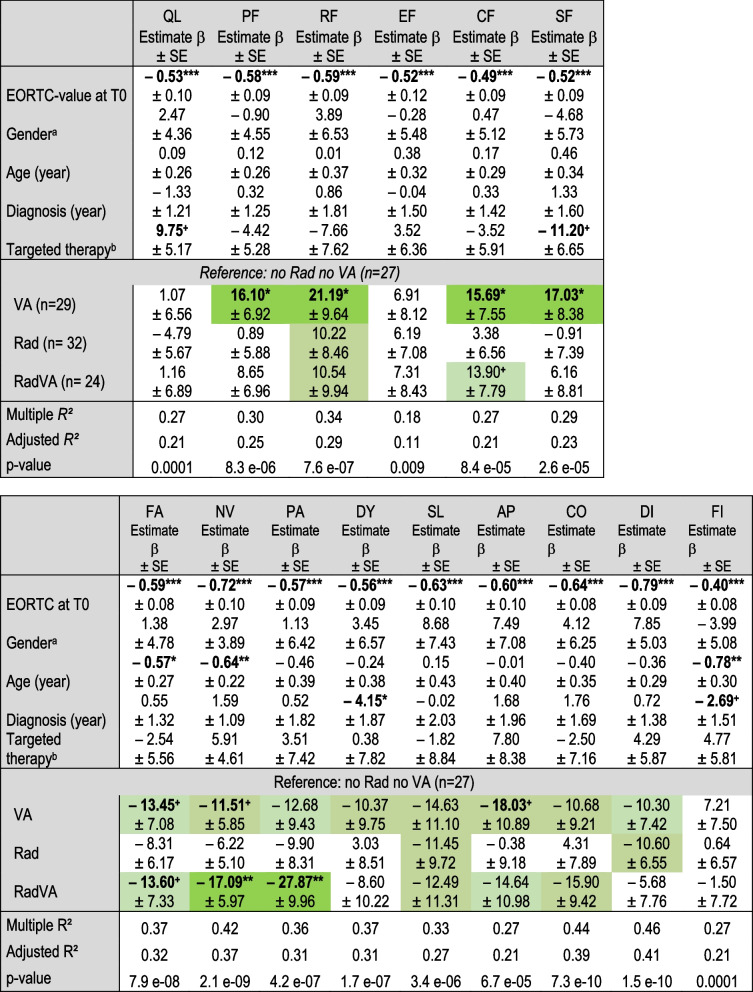
Association factors for EORTC QLQ-C30 changes

Multivariate linear regression analyses were performed for EORTC QLQ-C30 scale changes (T0– > T1). Improvements of functional scales are indicated by positive whereas symptoms by negative estimates. Improvements > 10 are highlighted in green

Significant p-values are indicated: ***p-value < 0.001; **p-value < 0.01;*p-value < 0.05; +p-value < 0.1

^a^Reference: male

^b^Reference: no targeted therapy

*SE* standard error; *QL* global health status; functional scales: *PF* physical; *RF* role; *EF* emotional; *CF* cognitive; *SF* social; symptoms: *FA* fatigue; *NV* nausea/vomiting; *PA* pain; *DY* dyspnea; *SL* insomnia; *AP* appetite loss; *CO* constipation; *DI* diarrhea; *FI* financial difficulties

For the combined RadVA patients significant and clinically relevant reductions in pain (estimate β = − 27.9 ± 10.0; p = 0.006) and nausea/vomiting (estimate β = − 17.1 ± 6.0; p = 0.005) were found with multivariate analyses with strong effect sizes (adjusted *R*^*2*^ > 0.30). Regarding confounding variables, significant associations were found between higher age and reduced fatigue (p = 0.04), nausea/vomiting (p = 0.005), and financial difficulties (p = 0.009). Furthermore, a significant association between the year of diagnosis (2013–2020) and a reduction in dyspnea (reduction of 4 points per increasing year; p = 0.03) was observed (Table 4).

## Discussion

The findings of the present RWD study reveal significant improvements of self-reported QoL in LC patients receiving VA therapy alone or in addition to radiotherapy.

Regarding QoL, it has been shown in clinical trials that VA treatment reduces chemotherapy-related side effects and improve tolerability, which then in turn may have a positive impact on QoL [9, 10]. As to the clinical outcome for cancer patients with respect to radiochemotherapy with add-on VA extracts the results have been primarily published for tumor stage I-III colorectal cancer patients [17] or locally advanced rectal cancer patients in a neoadjuvant setting [18]. The first publication indicated fewer therapy-related adverse events and a survival benefit for the combinational group and the latter publication described a better pathologic complete response for the combinational versus the control group (53.5% vs. 21.6%, p = 0.04).

In breast cancer patients it was previously described that several EORTC QLQ-C30 functioning as well as symptoms scales improved in association with concomitant VA therapy [26]. Similarly, in a RWD study, our group demonstrated the impairing effects of chemotherapy and the positive effects of VA applications on self-reported QoL parameters [27]. The data of the present study are in line with a randomized controlled trial of 223 cancer patients, of whom 94 were diagnosed with LC, improvements in self-reported fatigue, insomnia, loss of appetite, nausea, and pain, and a reduction in chemotherapy related side effects were reported in relation to concomitant VA therapy [16]. Also, in a randomized trial of 72 inoperable LC patients, adverse effects and QoL evaluations revealed some benefits for the VA group compared to the non-VA group [15].

In the present RWD study, marked, although not significant, reductions ranging from 10 to 18 points were observed for all the symptom scales except financial difficulties in patients receiving VA treatment without radiation (Table 4). Because limiting the number and severity of disease symptoms has been shown to be critical to improving the QoL in LC patients [8], this may explain why the trend of symptom reduction observed here appears to correlate with significantly favorable associations for physical, role, cognitive, and social functioning. It seems unlikely that the observed substantial QoL improvements among patients receiving add-on VA can be fully explained by natural history or regression to the mean. The present study reveals that with respect to the total study cohort global health and emotional functioning improved within the 12-month observation period, whereas the other functional scales, particularly physical functioning, were rather deteriorated (Table 3). However, for patients receiving VA treatment, significant improvements in physical, role, cognitive, and social functioning were detectable. It appears that good physical functioning is crucial, as a meta-analysis on QoL in LC patients found that physical activity in particular was associated with better global health, improved mood, and reduced symptom burden [28], indicating that VA applications may have a beneficial impact on QoL.

Cancer-related pain is multifactorial, and for that optimal pain relief multimodal treatments including anticancer therapies and analgesics should be implemented to achieve the best possible QoL. According to WHO recommendations, radiotherapy is used to reduce the need for analgesics and improve QoL [29]. In a recent report evaluating the impact of curative radiotherapy on QoL, data from 510 treated LC patients showed no significant impact of radiotherapy on QoL changes [30]. Also in our study, we observed no significant QoL-changes with radiotherapy alone.

Even though patients in the combined RadVA group had more advanced cancer stages than the other groups (Table 1), pronounced significant reductions in pain symptoms and nausea/vomiting were observed. These positive findings support the potential efficacy of combined RadVA therapy in regard to QoL.

Limitations of our study include the non-randomized, non-controlled, and unblinded nature of the study design which is prone to various biases including selection bias. A possible bias arises from the fact that the observation period was 12 months and therefore no conclusions can be drawn about the QoL of patients who were unable or unwilling to respond after 12 months, or who were already deceased. However, we tried to reduce confounding bias by conducting adjusted multivariate linear regression analyses. Nevertheless, this RWD study provides implications for the clinical efficacy of concomitant VA treatment for LC patients being consistent with published data in LC patients and for other cancer entities. The strengths of our study include the presentation of real-world care of LC patients in a German certified lung cancer center.

## Conclusions

Our results suggest that self-reported QoL has been improved 12 months after diagnosis in lung cancer patients receiving VA therapy. A remarkable benefit on self-reported pain, nausea and vomiting appears to be associated with combined radiation and VA treatment in this cohort. This stresses the importance of considering and evaluating combined oncological treatments with VA in future concepts for the improvement of QoL in lung cancer patients.

## Data Availability

The anonymized data that support the findings of this study are openly available in the repository figshare.com (https://figshare.com/articles/dataset/QoL_Lung/20330397) all other information will be available on reasonable request.

## Abbreviations

DRKS: Deutsches Register Klinischer Studien
EORTC QLQ-C30: European Organization of Research and Treatment Health-Related Quality of Life Core Questionnaire scale
GKH: Gemeinschaftskrankenhaus Havelhöhe
IQR: Interquartile range
LC: Lung cancer
N: Numbers
NO: Network Oncology
NSCLC: Non-small-cell lung cancer
QoL: Quality of life
Rad: Radniation
RWD: Real-World-Data
SCLC: Small-cell lung cancer
UICC: Union for International Cancer Care
VA: *Viscum album* L.

## References

[1] Siegel RL, Miller KD, Fuchs HE, Jemal A (2022). Cancer statistics, 2022. CA Cancer J Clin.

[2] Bennett BM, Wells JR, Panter C, Yuan Y, Penrod JR (2017). The humanistic burden of small cell lung cancer (SCLC): a systematic review of health-related quality of life (HRQoL) literature. Front Pharmacol.

[3] Poghosyan H, Sheldon LK, Leveille SG, Cooley ME (2013). Health-related quality of life after surgical treatment in patients with non-small cell lung cancer: a systematic review. Lung Cancer.

[4] S3-Leitlinie. S3-leitlinie prävention, diagnostik, therapie und nachsorge des lungenkarzinoms. Leitlinienprogramm Onkologie [Internet]. 2022 2022; (Version 2.1—Dezember 2022):[658 p.].

[5] Langendijk JA, Aaronson NK, de Jong JM, ten Velde GP, Muller MJ, Lamers RJ (2001). Prospective study on quality of life before and after radical radiotherapy in non-small-cell lung cancer. J Clin Oncol Off J Am Soc Clin Oncol.

[6] Slotman BJ, Mauer ME, Bottomley A, Faivre-Finn C, Kramer GWPM, Rankin EM (2009). Prophylactic cranial irradiation in extensive disease small-cell lung cancer: short-term health-related quality of life and patient reported symptoms—results of an international phase iii randomized controlled trial by the EORTC radiation oncology and lung cancer groups. J Clin Oncol.

[7] Steinmann D, Paelecke-Habermann Y, Geinitz H, Aschoff R, Bayerl A, Bölling T (2012). Prospective evaluation of quality of life effects in patients undergoing palliative radiotherapy for brain metastases. BMC Cancer.

[8] Polanski J, Jankowska-Polanska B, Rosinczuk J, Chabowski M, Szymanska-Chabowska A (2016). Quality of life of patients with lung cancer. Onco Targets Ther.

[9] Kienle GS, Kiene H (2010). Review article: influence of *Viscum album* L. (European mistletoe) extracts on quality of life in cancer patients: a systematic review of controlled clinical studies. Integr Cancer Ther.

[10] Loef M, Walach H (2020). Quality of life in cancer patients treated with mistletoe: a systematic review and meta-analysis. BMC Complement Med Ther.

[11] Schad F, Thronicke A, Steele ML, Merkle A, Matthes B, Grah C (2018). Overall survival of stage IV non-small cell lung cancer patients treated with *Viscum album* L. in addition to chemotherapy, a real-world observational multicenter analysis. PLoS ONE.

[12] Bouazza YB, Chiairi I, El Kharbouchi O, De Backer L, Vanhoutte G, Janssens A (2017). Patient-reported outcome measures (PROMs) in the management of lung cancer: a systematic review. Lung Cancer.

[13] Wood R, Taylor-Stokes G, Smith F, Chaib C (2019). The humanistic burden of advanced non-small cell lung cancer (NSCLC) in Europe: a real-world survey linking patient clinical factors to patient and caregiver burden. Qual Life Res Int J Qual Life Asp Treat Care Rehabil.

[14] Thronicke A, von Trott P, Kröz M, Grah C, Matthes B, Schad F (2020). Health-related quality of life in patients with lung cancer applying integrative oncology concepts in a certified cancer centre. Evid-Based Complement Altern Med eCAM.

[15] Bar-Sela G, Wollner M, Hammer L, Agbarya A, Dudnik E, Haim N (2013). Mistletoe as complementary treatment in patients with advanced non-small-cell lung cancer treated with carboplatin-based combinations: a randomised phase II study. Eur J Cancer.

[16] Piao BK, Wang YX, Xie GR, Mansmann U, Matthes H, Beuth J (2004). Impact of complementary mistletoe extract treatment on quality of life in breast, ovarian and non-small cell lung cancer patients. A prospective randomized controlled clinical trial. Anticancer Res.

[17] Friedel WE, Matthes H, Bock PR, Zanker KS (2009). Systematic evaluation of the clinical effects of supportive mistletoe treatment within chemo- and/or radiotherapy protocols and long-term mistletoe application in nonmetastatic colorectal carcinoma: multicenter, controlled, observational cohort study. J Soc Integr Oncol.

[18] Baek J-H, Jeon Y, Han K-W, Jung DH, Kim K-O (2021). Effect of mistletoe extract on tumor response in neoadjuvant chemoradiotherapy for rectal cancer: a cohort study. World J Surg Oncol.

[19] Schad F, Axtner J, Happe A, Breitkreuz T, Paxino C, Gutsch J (2013). Network oncology (NO)–a clinical cancer register for health services research and the evaluation of integrative therapeutic interventions in anthroposophic medicine. Forschende Komplementarmedizin.

[20] Thronicke A, Oei SL, Merkle A, Herbstreit C, Lemmens HP, Grah C (2018). Integrative cancer care in a certified cancer centre of a German anthroposophic hospital. Complement Ther Med.

[21] Aaronson NK, Ahmedzai S, Bergman B, Bullinger M, Cull A, Duez NJ (1993). The European organization for research and treatment of cancer QLQ-C30: a quality-of-life instrument for use in international clinical trials in oncology. J Natl Cancer Inst.

[22] Fayers PM, Aaronson NK, Bjordal K, Groenvold M, Curran D, Bottomley A, et al. The EORTC QLQ-C30 scoring manual (3rd Edition) Brüssel: European organisation for research and treatment of cancer; 2001 [updated 2018; cited 2001].

[23] Cohen J (1992). A power primer. Psychol Bull.

[24] Team RC. R: A language and environment for statistical computing 2016.

[25] Scott NW, Fayers PM, Aaronson NK, Bottomley A, Group EQoL. EORTC-QLQ-C30 reference values. https://www.eortc.org/app/uploads/sites/2/2018/02/reference_values_manual2008.pdf The EORTC Quality of Life Group; 2008 [cited 2008].

[26] Tröger W, Zdrale Z, Tišma N, Matijašević M (2014). Additional therapy with a mistletoe product during adjuvant chemotherapy of breast cancer patients improves quality of life: an open randomized clinical pilot trial. Evid-Based Complement Altern Med eCAM.

[27] Oei SL, Thronicke A, Kröz M, von Trott P, Schad F, Matthes H (2020). Impact of oncological therapy and *Viscum album* L. treatment on cancer-related fatigue and internal coherence in nonmetastasized breast cancer patients. Integr Cancer Ther.

[28] Teba PP, Esther MG, Raquel SG. Association between physical activity and patient-reported outcome measures in patients with lung cancer: a systematic review and meta-analysis. Q Life Res Int J Q Life Asp Treatme Care Rehabil. 2022 Jan 21. PubMed PMID: 35059948. Epub 2022/01/22. eng. 10.1007/s11136-021-03053-3 35059948

[29] WHO-guideline. WHO guidelines for the pharmacological and radiotherapeutic management of cancer pain in adults and adolescents. Geneva, Switzerland: WHO, 2018. 30776210

[30] Van der Weijst L, Aguado-Barrera ME, Azria D, Berkovic P, Boisselier P, Briers E (2022). Overview of health-related quality of life and toxicity of non-small cell lung cancer patients receiving curative-intent radiotherapy in a real-life setting (the REQUITE study). Lung Cancer.

